# On the occurrence of mirror modes at Mercury and their comparison with magnetic holes and ion cyclotron waves

**DOI:** 10.1186/s40623-026-02533-5

**Published:** 2026-10-08

**Authors:** Martin Volwerk, Tomas Karlsson, Charlotte Goetz, Daniel Heyner, Ferdinand Plaschke, Daniel Schmid, Cyril Simon Wedlund, Rumi Nakamura, Francesco Califano, Maria Hamrin, Francesco Pucci, Adriana Settino, Diana Rojas-Castillo, Georg Blüthner

**Affiliations:** 1 Space Research Institute, Austrian Academy of Sciences, Graz, Austria; 2 Department of Space and Plasma Physics, School of Electrical Engineering and Computer Science, Royal Institute of Technology, Stockholm, Sweden; 3 Department of Mathematics, Physics and Electrical Engineering, Northumbria University, Newcastle upon Tyne, NE1 8ST UK; 4 Institute for Geophysics and Extraterrestrial Physics, Technische Universität Braunschweig, Braunschweig, 38106 Germany; 5 Institute of Physics, University of Graz, Graz, Austria; 6 Department of Physics, University of Pisa, Pisa, Italy; 7 Department of Physics, Umeå University, Umeå, Sweden; 8 Istituto per la Scienza e la Tecnologia dei Plasmi, ISTP, Bari, Italy; 9 Space Physics Group, Geophysics Institute, National Autonomous University of Mexico, Mexico City, Mexico

**Keywords:** MESSENGER, Magnetometer, Mirror modes

## Abstract

The MESSENGER magnetometer data for the years 2011 through 2015 are used to study the presence of mirror modes in Mercury’s environment. Using magnetic-field-only-detection of these structures, 13,691 intervals of 20 s and longer are found in the solar wind, and in the magnetosheath and magnetosphere of Mercury. The distribution of mirror modes around the planet is similar to that found at Venus and Mars, however, the occurrence rate of $\sim \, 1.0\, \times \,10^{-4}$ per second is up to 2 orders of magnitude smaller than at the other two planets. The mirror modes are more prone to be excited near Mercury’s aphelion. A comparison shows that mirror modes and magnetic holes have a similar behaviour around Mercury, and that about 40% of the mirror modes can also be identified as magnetic holes. As ion cyclotron waves can be generated by the same temperature anisotropy, they are also compared with the mirror modes. It is found that the ion cyclotron waves show a lesser dependence on the true anomaly angle.

**Graphical Abstract:**

**Figure:**
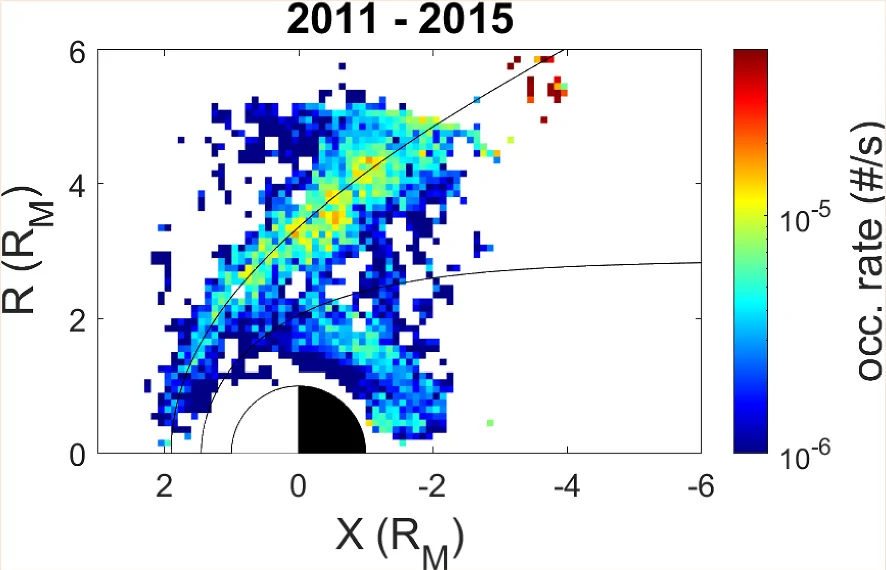

Occurrence rate of mirror modes around Mercury for the whole MESSENGER mission

## Introduction

Mirror modes (MiMos) are ubiquitous structures in space plasmas. They are characterized by depressions of the magnetic field strength and increases in the plasma pressure, or vice versa as peaks in the magnetic field strength and a dip in plasma pressure (Southwood and Kivelson 1993; Kivelson and Southwood 1996). These structures have been found in the magnetosheaths of the Earth (Tsurutani et al. 1982; Baumjohann et al. 1999; Lucek et al. 1999a, b; Soucek et al. 2015) and in its magnetotail (Nishida et al. 1997; Ge et al. 2011), the magnetosheath of Venus (Volwerk et al. 2008, 2016, 2023), of Mars (Simon Wedlund et al. 2022, 2023, 2025), of Jupiter (Joy et al. 2006), of Saturn (Erdös and Balogh 1996; Bavassano Cattaneo et al. 1998) and at comets (Glassmeier et al. 1993; Schmid et al. 2014; Tello Fallau et al. 2023).

## Instability criteria for MiMos

MiMos are created by a temperature anisotropy in the plasma (Hasegawa 1969; Gary 1992; Gary et al. 1993) with the temperature perpendicular to the magnetic field sufficiently greater than along the magnetic field.

Hasegawa (1969) showed that in a bi-maxwellian multi-component plasma the Mirror Mode Instability Criterion (MMIC) is:

1 $$\begin{aligned} \mathrm{MMIC} = 1 + \sum _i \beta _{i\perp } \left( 1 - \frac{T_{i\perp }}{T_{i\Vert }} \right) < 0, \end{aligned}$$

with $T_{i,\perp }$ the temperature perpendicular to the background magnetic field and $T_{i,\Vert }$ the temperature parallel to it and

2 $$\beta _{i,\perp } = \frac{n_i k_\mathrm{B} T_{i\perp }}{B^2/2\mu _0}$$

is the perpendicular plasma-β of species *i*, the ratio of perpendicular (to the magnetic field) plasma pressure and the magnetic pressure. Here $n_i$ is the number density of species *i*, $k_\mathrm{B}$ is Boltzmann’s constant and $\mu _0$ is the permeability of vacuum. This instability criterion, Eq. (1), actually gives rise to two different kinds of waves, the Alfvén ion cyclotron wave and the mirror mode, depending on the plasma-β.

**Fig. 1:**
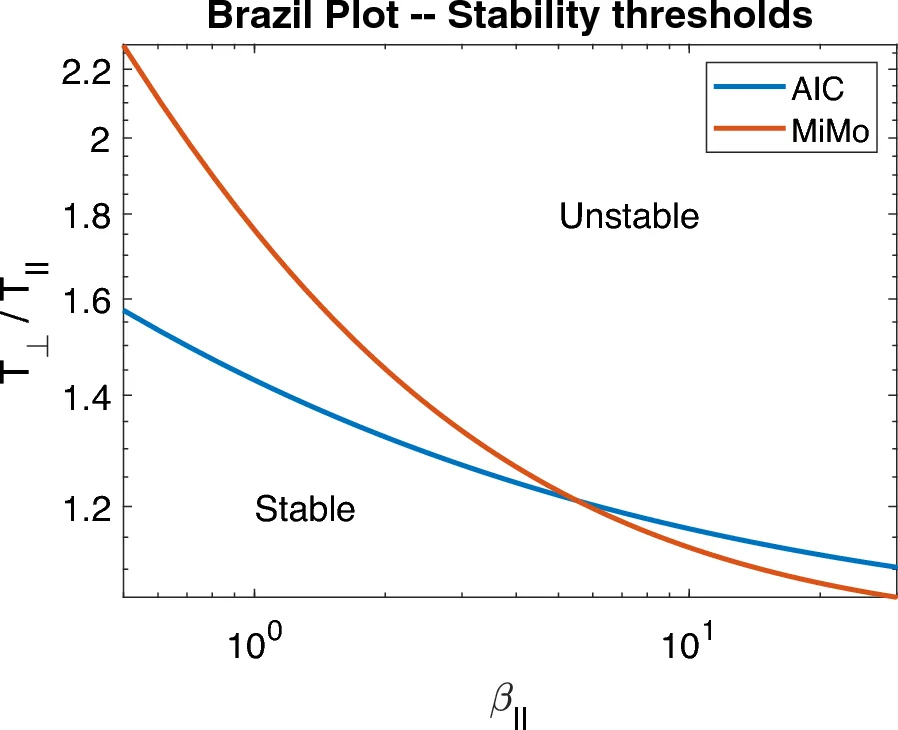
The stability thresholds for the (Alfvénic) Ion Cyclotron (AIC) and Mirror Mode (MiMo) instabilities as a function of the parallel plasma-β and the ratio of perpendicular and parallel temperature in a reduced Brazil plot. The curves are described by $T_{\perp }/T_{\Vert } = 1 + (a / (\beta _{\Vert } - \beta _0)^b)$. The parameters *a*, *b* and $\beta _0$ are given by Hellinger et al. (2006). AIC: a=0.43, b=0.42, $\beta _0 = -0.0004$. MiMo: a=0.77, b=0.76, $\beta _0 = 0.016$. The curves are calculated for a weakly magnetized plasma with $\omega _\mathrm{pe}/\omega _\mathrm{ce} = 100$ and $\gamma _\mathrm{max} = 10^{-3} \omega _\mathrm{cp}$

Another approach to the determination of the instability criteria for ion-scale MiMos was presented by Pokhotelov et al. (2004) and Hellinger et al. (2006). They use the fully kinetic theory for high-β plasmas and finite ion Larmor radius effect to model the mirror and Alfvén ion cyclotron instabilities and find that the approximate threshold of these can be fitted with the following relation:

3 $$\begin{aligned} \frac{T_{\perp p}}{T_{\Vert p}} = 1 + \frac{a}{\beta _{\Vert p}^b}, \end{aligned}$$

for marginal stability, i.e. the maximum growth rate $\gamma _\mathrm{max} \sim \, 0$. The values for *a* and *b* are obtained by fitting. For $\gamma _\mathrm{max} = 10^{-3} \omega _{cp}$, Samsonov et al. (2001) find that the cyclotron instability has a≃0.45 and b≃0.40, and the MiMo instability a≃0.74 and b≃0.73. Hellinger et al. (2006) further develops this relationship to:

4 $$\begin{aligned} \frac{T_{\perp p}}{T_{\Vert p}} = 1 + \frac{a}{(\beta _{\Vert p} - \beta _0)^b}, \end{aligned}$$

which is used in Fig. 1. The same relationship can be used to describe the instability curves for parallel and oblique firehose instabilities. This kind of analysis usually leads to a so-called Brazil plot, of which only the upper part, i.e. $T_{\perp }/T_{\Vert } > 1$ is shown in Fig. 1, in which the magnetoplasma measurements are placed to investigate its stability.

For large values the mirror mode instability dominates, for small values the ion cyclotron instability (see Fig. 1). However, for both instabilities a larger plasma-β makes it easier for them to develop, i.e. at lower $T_{\perp }/T_{\Vert }$.

The generation of the temperature anisotropy around Mercury (or other solar system bodies) can be facilitated by pick-up of freshly created ions in the exosphere, injecting a ring–beam distribution into the solar wind plasma, which is unstable for the generation of both mirror modes and ion cyclotron waves. Another way is by the solar wind plasma crossing the quasi-perpendicular bow shock, which will mainly increase the perpendicular temperature of the ions, and thereby generate mirror modes. 1

Recent analysis of magnetometer data by Schmid et al. (2021, 2022) showed that there are waves observed near the ${\textbf{H}^{+}}$ gyrofrequency, often referred to as Ion Cyclotron Waves (ICWs) in the Hermean exosphere, as well as evidence for ${\textbf{H}_{2}^{+}}$ and ${\textbf{He}^{+}}$ (Weichbold et al. 2025a, b) and even ${\textbf{Li}^{+}}$ (Schmid et al. 2025). These waves are created by the pick-up of freshly created ions in Mercury’s exosphere. The picked-up ions generate a ring–beam distribution in the particle distribution function, creating the temperature anisotropy driving the instability. The ring–beam, moving sunward, will Doppler-shifted interact with the plasma turbulent spectrum: $\omega - \textbf{k}\cdot \textbf{v}_\mathrm{sw} = \pm n\Omega _i$ (with usually n=1). The phase velocity of the waves is assumed to be the Alfvén speed, which is much slower than the solar wind speed, and thus are transported anti-sunward and observed Doppler-shifted backwards by the spacecraft, ending up with the local gyro frequency. This is the so-called anomalous Doppler shift (Mazelle and Neubauer 1993). Indeed, for the heavier pick-up species one needs to be careful not to be in the foreshock region, where reflected protons could interact with the solar wind in a beam–beam interaction, leading to Doppler shifted waves in the frequency range of the ion cyclotron frequencies of heavier ions. The exclusion of the foreshock is discussed in, e.g., Weichbold et al. (2025b). Simon Wedlund et al. (2025) have shown that pick-up ions can also generate MiMos at Mars.

The presence of MiMos in the magnetic environment of Mercury was predicted by numerical simulations (Herčik et al. 2013). However, using MESSENGER data to investigate waves in the Hermian magnetosheath, Sundberg et al. (2015) uses a searching method that requires the waves to be coherent, which will be unfavourable for the detection of MiMos. They state that, after a first overview, the MiMos seem to be relatively rare in the Hermean magnetosheath. Up to now, no measurements of MiMos around Mercury have been published. Another structure, related to the MiMo, is the Magnetic Hole (MH), which is usually a singular depression of the magnetic field with increased plasma pressure (Volwerk et al. 2021). It is sometimes assumed that MHs are the end stage of MiMos, as they are often present in MiMo-stable plasmas (Winterhalter et al. 1995). Karlsson et al. (2021) have detected the presence of MHs in the Hermean magnetoenvironment, and have shown that they can cross the bow shock and enter the magnetosheath (see also Karlsson et al. 2022). They also show that the discrimination between MH and MiMo can be difficult. Trains of magnetic depressions were observed both in the solar wind and in the Hermean magnetosheath. Using 4 Earth-years of data they found 2,154 isolated magnetosheath MHs, which corresponds to an occurrence rate of 17 per day or ${2\times 10^{-4}}{\#/\text {s}}$, and 2726 isolated solar wind MHs, corresponding to 4.4 per day or ${5\times 10 ^{-5}}{\#/\text {s}}$. Isolated means that, within ${\pm \,60\,\mathrm{s}}$ around the time of MH observation, no other MH was present. Observations of multiple MHs can also be interpreted as MiMos (see discussion below). Lately, numerical investigations have shown that the relaxation of turbulent fluctuations can generate multiple magnetic holes (Arró et al. 2024). This shows a second path for the generation of MHs that is not related to the relaxation or the non-linear saturation of the mirror instability.

In this paper, we investigate the presence of MiMos in the Hermean environment. We concentrate on their occurrence rate, location around Mercury and dependence on season and True Anomaly Angle (TAA^2^). We also compare our results with the observations of MHs and ICWs.

## Data analysis

In this study, the MESSENGER (MErcury Surface, Space ENvironment, GEochemistry, and Ranging, Solomon et al. 2007) spacecraft magnetometer data ( Anderson et al., 2007) are used at a resolution of 1 s. We also use the Fast Imaging Plasma Spectrometer (FIPS) data (Raines et al. 2011), which have a varying resolution, with highest rate of 10 s. It should be noted that the MESSENGER spacecraft contains a heat-shield behind which the instruments are protected from the solar radiation. This means that FIPS only has a limited view of the ambient plasma, which results in a strongly diminished count rate and thus ion density. However, this can be used as a proxy for the local plasma density, but not for a study of pressure balance over the structures.

The identification of the MiMos is performed with magnetic field data only (see e.g., Lucek et al. 1999a; Volwerk et al. 2008; Simon Wedlund et al. 2022; Schmid et al. 2014; Tello Fallau et al. 2023). This method may lead an ambiguity with slow-mode waves. However, for missions at which plasma data are available at an appropriate temporal resolution, it has been shown that this method works well at identifying MiMos (see, e.g., Rae et al. 2007; Soucek et al. 2008). The magnetic field data, $\textbf{B}$, are low-pass filtered for waves with periods longer than 3 min, which is used as the background field, $\textbf{B}_\mathrm{bk}$. The 3 min interval is an empirically determined value, which has worked well in earlier papers on MiMos and MHs (Volwerk et al. 2016, 2020; Karlsson et al. 2021; Volwerk et al. 2021; Simon Wedlund et al. 2023). The variations in the field magnitude, $B_\mathrm{m}$, with respect to the background field magnitude, $B_\mathrm{bk}$, are determined by:

5 $$\begin{aligned} B_\mathrm{var} = \frac{\Delta B}{B_\mathrm{bk}} = \frac{|B_\mathrm{m} - B_\mathrm{bk}|}{B_\mathrm{bk}}. \end{aligned}$$

A sliding window of 20 s, with a time shift of 1 s, is then used for minimum variance analysis (Sonnerup and Scheible 1998) to determine the eigenvalues, λ, the eigenvectors, and the angle between minimum(maximum) variance direction and $\textbf{B}_\mathrm{bk}$, $\theta _\mathrm{Bmv}(\phi _\mathrm{Bmv})$, as well as the elevation (*el*) and azimuth (*az*) of the magnetic field in the MSO^3^ coordinate system. Then the following criteria are put onto these quantities (see also Table 1 in Simon Wedlund et al. 2022):

- $B_\mathrm{var} > 0.15$ (Génot et al. 2008);
- $\theta _\mathrm{Bmv} < 20^{\circ }$ (Price et al. 1986; Lucek et al. 1999b);
- $\phi _\mathrm{Bmv} > 70^{\circ }$ (Volwerk et al. 2008);
- $\lambda _\mathrm{max} / \lambda _\mathrm{int} \ge 3$, (linear polarization of structure, Génot et al. 2001; Soucek et al. 2008);
- $\lambda _\mathrm{int} / \lambda _\mathrm{min} \le 8$, (linear polarization of structure, Génot et al. 2001; Soucek et al. 2008).

## Results

**Fig. 2:**
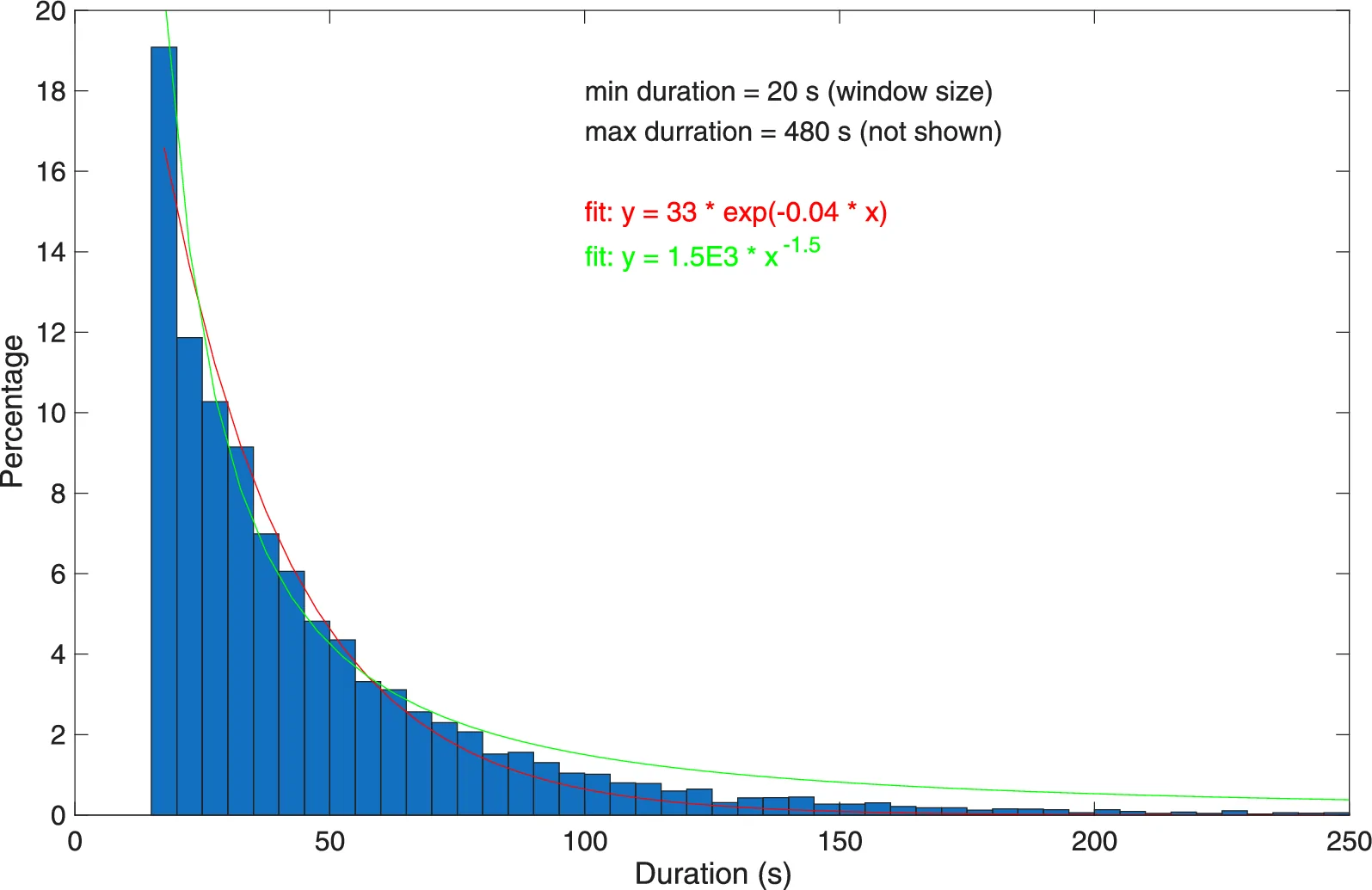
Histogram of the duration of the MiMo events in percentage of the total number of events (13.691) in bins of 5 s width

For the years 2011 through 2015, we have determined the intervals which agree with the magnetic field criteria for MiMos, as described in the previous section. Here, we assume that events that are separated by less than 30 s are one event (see also Volwerk et al. 2008). Over these four years a total of 13,691 events were found varying in duration from 20 s (the sliding window size) to 480 s. The distribution of duration is shown in Fig. 2. In general, we find an overall occurrence rate of 7 per day, smaller than the MH occurrence rate of 17 per day in the magnetosheath but larger than the 4.4 per day for isolated MHs in the solar wind (Karlsson et al. 2021). The occurrence rates per environment (solar wind, magnetosheath, magnetosphere) of the MiMos will be shown later in Sect. 5. The criteria on the structures in this paper, on themselves, do not discern between solar wind, magnetosheath or magnetosphere. However, Philpott et al. (2020) presented a list of boundary crossings for the MESSENGER mission, which we use to determine in which region each event was detected.

### Events

**Fig. 3:**
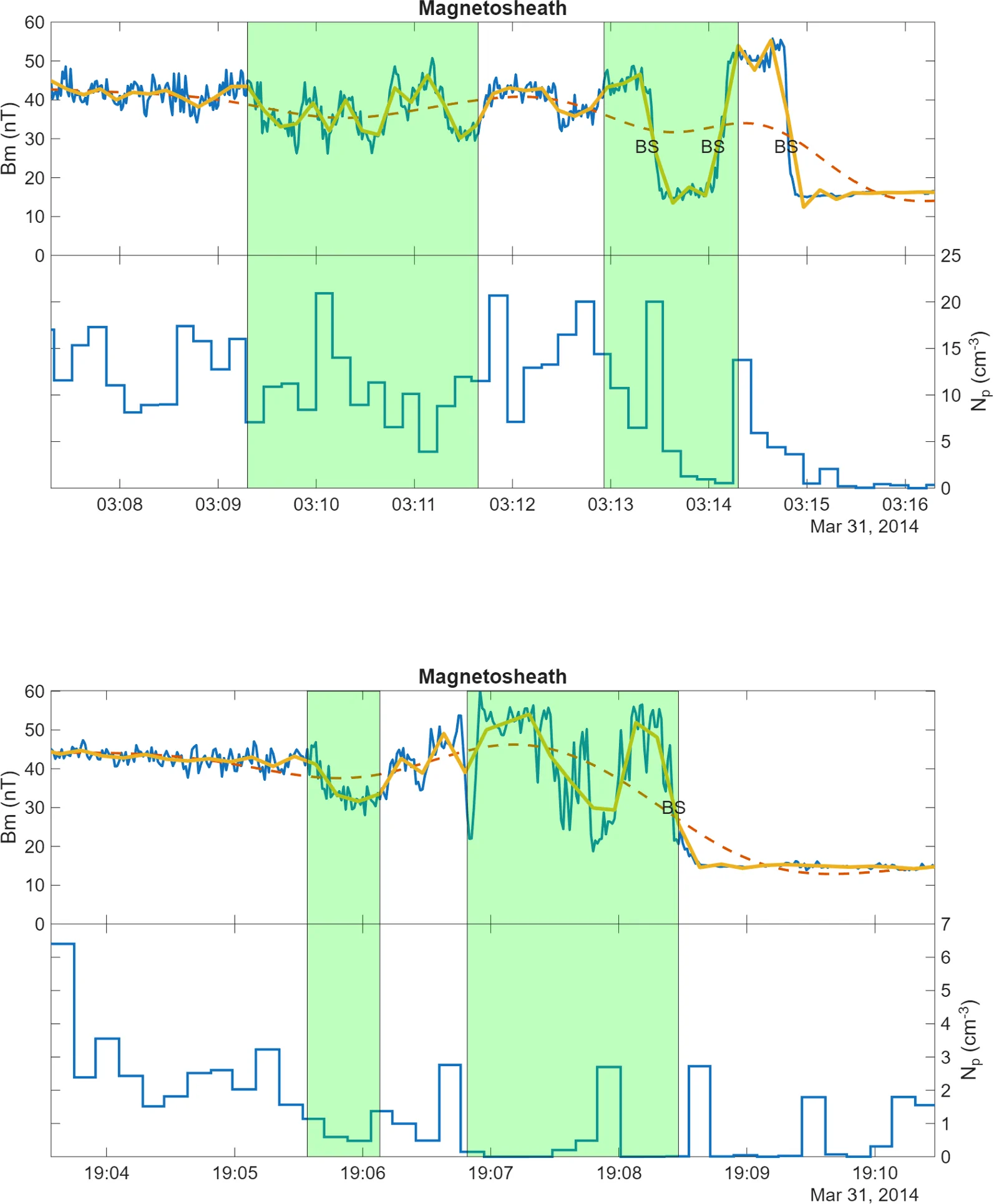
Two intervals on 31 March 2014 with MiMos behind a quasi-perpendicular bow shock. The top panels show the total magnetic field strength (solid blue) and the background field strength ($B_\mathrm{bk}$ dashed red) and the resampled magnetic field at a rate of 0.1 Hz similar as the FIPS data resolution (yellow), the bottom panels show the proton density. The green-shaded areas show the selected MiMo events. Around 03:10 UT there is an anti-correlation between field strength and proton density. The event around 03:14 UT has the magnetic characteristics of a MiMo, but the proton density drops sharply too. As can be seen from the plot, this event rather corresponds to the bow shock moving in and out over the spacecraft. The events at 19:06 UT and 19:08 UT show magnetic depressions combined with possible increases in proton density

As mentioned above, the identification of the regions in which the events discussed here are located is based on the list by Philpott et al. (2020). Examples of MiMos at the dusk-side magnetosheath, near $(1.3, 2.3, -\,0.7) \, R_\mathrm{M}$,^4^ behind a quasi-perpendicular bow shock, $\theta _\mathrm{BN} \approx 65^{\circ }$, are shown in Fig. 3: the total magnetic field data, $B_\mathrm{m}$ (solid blue), the background field $B_\mathrm{bk}$ (dashed red), and the proton density, $N_\mathrm{p}$. The green-shaded intervals show the regions where the magnetic MiMo criteria are fulfilled. It is clear that the resolution of the plasma data is too coarse to resolve the smaller MiMos.

In the first green-shaded interval in the top plot of Fig. 3 (03:07–03:16 UT), there is an anti-correlation between the magnetic field strength and the proton density, as expected for MiMos. Interestingly, the magnetic field first shows dips with respect to the background field and at the end shows peaks. This is coarsely reflected also, in anti-phase, in the plasma density. In the second green-shaded interval (19:03:30–19:10:30 UT) there is a broad dip in the magnetic field strength, which is accompanied by a decrease in the proton density. This is, most likely, the BS moving in and out. See also the bottom panel in Fig. 3, where at the end we see a very quiet magnetic field at 15 nT and low density, indicative of the solar wind.

The bottom panel in Fig. 3 shows MESSENGER data also in the Hermean magnetosheath, with two MiMo intervals, where the plasma instrument captures the largest dips, albeit slightly shifted in the first green-shaded interval due to the integration time of the instrument.

**Fig. 4:**
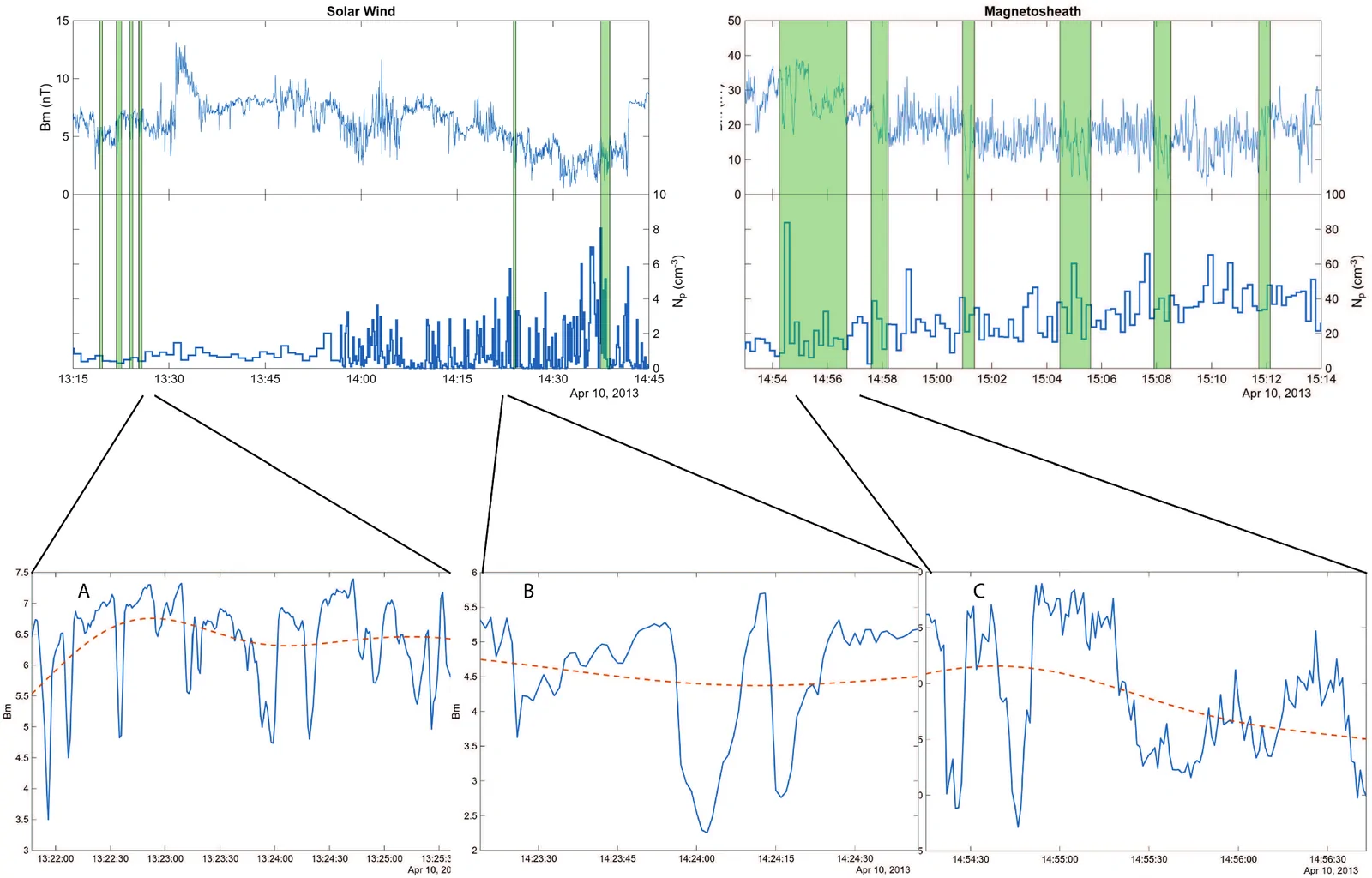
Two intervals on 10 March 2013 with MiMos in the solar wind and behind a quasi-parallel bow shock, in the same format at Fig. 3 but longer intervals. The bottom row of panels shows zoom-ins on MiMos. Panel A shows a train of MiMos in the solar wind. Panel B shows two MiMos in the solar wind. Panel C shows MiMos behind the quasi-parallel bow shock

In Fig. 4 we show two periods on 10 April 2013 where MESSENGER moves from the solar wind into the Hermean magnetosheath, from $\sim \, (-\,0.8, -\,0.8, -\,4.8) \, R_\mathrm{M}$ to $\sim \, (-\,1.0, -\,2.1, -\,2.6) \, R_\mathrm{M}$. The location, together with the solar wind magnetic field shows that the spacecraft crosses a quasi-parallel bow shock with $\theta _\mathrm{Bn} \approx 34^{\circ }$.

**Fig. 5:**
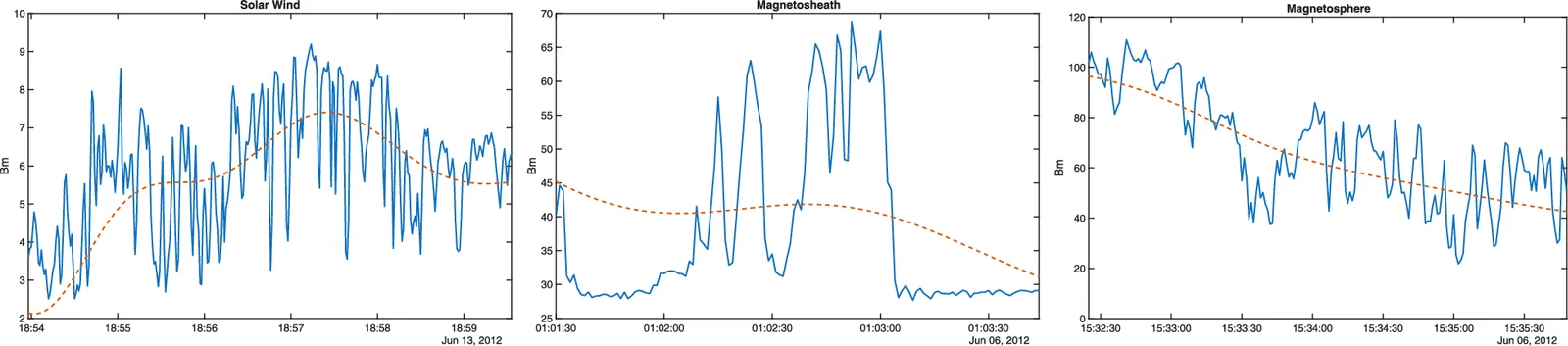
Three MiMo intervals in different regions. In the solar wind there is a long train of depressions. In the magnetosheath there is an example of MiMos in the form of peaks in the magnetic field strength. In the magnetosphere there is a train of irregular mirror modes

We zoom in onto three events in the bottom panels which show clear MiMOs:

A A series of magnetic depressions with varying widths of $\sim \, 6$ to ${\sim \, 10\,\mathrm{s}}$ is observed in the magnetometer data, which satisfy the MiMo criteria.
B A slightly extended interval around an identified MiMo at 14:24 UT, showing two large magnetic depressions with widths of ${\sim \, 16\,\mathrm{s}}$ and ${\sim \, 12\,\mathrm{s}}$, respectively.
C Magnetic field data measured in the Hermean magnetosheath, showing two MiMos with widths of ${\sim \, 9\,\mathrm{s}}$ and ${\sim \, 14\,\mathrm{s}}$, respectively.

A few other examples of MiMos are shown in Fig. 5, with a long train of magnetic depressions in the solar wind, MiMos in the form of peaks in the magnetic field strength in the magnetosheath and a train of irregular MiMos in the magnetosphere.

### Occurrence rate

**Fig. 6:**
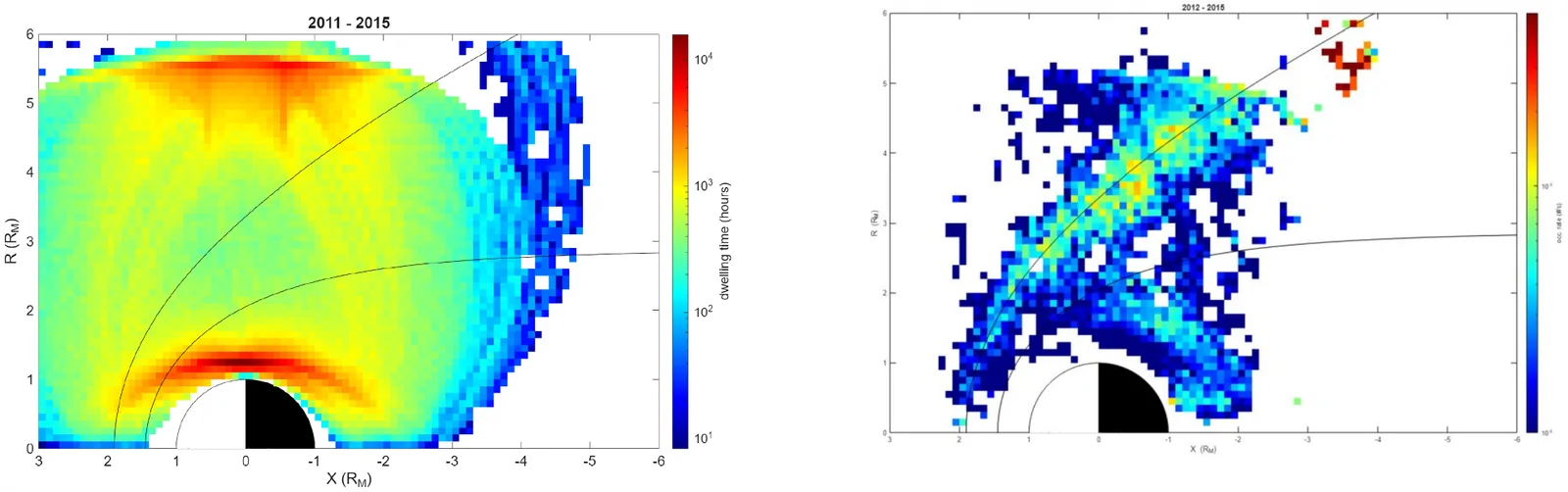
The dwell time of MESSENGER (left) and the occurrence rate of all MiMos (right) plotted around Mercury in cylindrical coordinates. The two drawn black lines are models of the bow shock (Slavin et al. 2009) and magnetopause (Shue et al. 1997). The highest occurrence rates (in $\#/\text {s}$) are along the bow shock and towards the magnetopause and behind the planet. The dwell times for the three regions are: solar wind 726.9 days, magnetosheath 201.5 days and magnetosphere 185.0 days

The occurrence rate of MiMos around Mercury is displayed in Fig. 6 similar to Volwerk et al. (2008) and Simon Wedlund et al. (2023). The events are collected in $0.1 \times 0.1 \, R_\mathrm{M}$ bins in *XR*-space (where $R = \sqrt{Y_\mathrm{MSO}^2 + Z_\mathrm{MSO}^2}$), for which also the dwell time of MESSENGER is determined. The occurrence rate is then shown as number per second ($\#/\text {s}$). There are three main locations where MiMos are prone to occur: 1. along the bow shock, both in the solar wind and in the magnetosheath (see Sect. 5); and 2. near the magnetopause, although not as pronounced, both in the magnetosheath and in the magnetosphere (see Sect. 5), and 3. curving behind the planet beyond the terminator. Note that the localized maximum overall occurrence rate is around ${\sim \, 1.e-5\,\mathrm{\#/\text {s}}}$, which is smaller than for Venus ${\sim \, 1.e-3\,\mathrm{\#/\text {s}}}$ (Volwerk et al. 2016) and Mars ${\sim \, 3.e-3\,\mathrm{\#/\text {s}}}$ (Simon Wedlund et al. 2023). The spatial distribution of the MiMos around the planet, however, is quite similar.

**Fig. 7:**
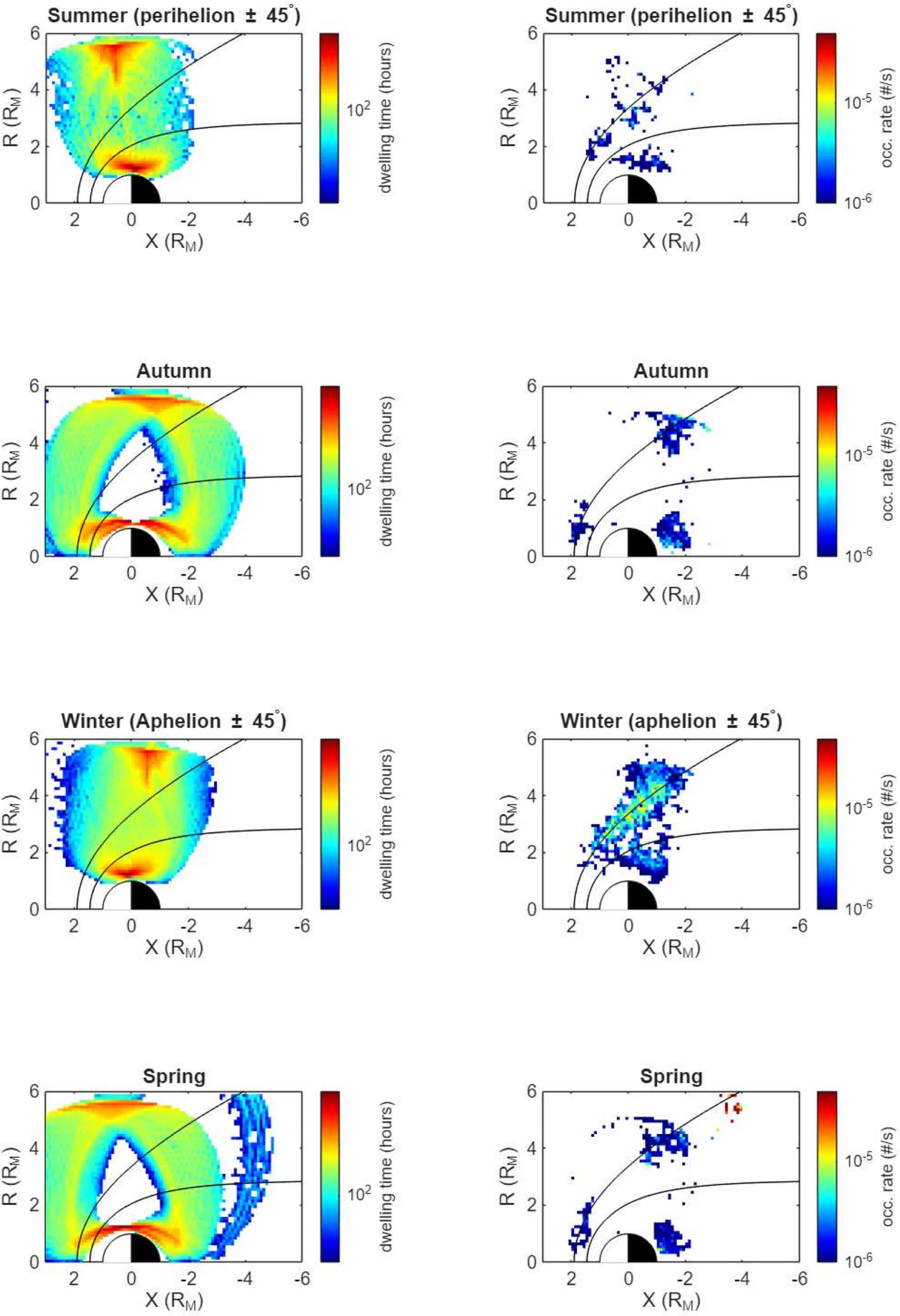
The dwelling time of Messenger around Mercury and the occurrence rate of MiMos separated by season (same format as Fig. 6). Table 1 shows the summed values

Mercury has a strongly eccentric orbit around the Sun with perihelion at ${\sim \, 0.31\,\mathrm{au}}$ and aphelion at ${\sim \, 0.47\,\mathrm{au}}$, which causes the seasons on the planet and the seasonal variation in the sodium exosphere (Cassidy et al. 2016) with higher column densities near aphelion. We have split up the orbit of Mercury in four seasons, and calculated the occurrence rate of MiMos for each one, as shown in Fig. 7. Interestingly, most of the MiMos are found during the Hermean winter season (aphelion $\pm 45^{\circ }$): Winter—6,116; Spring—2,982; Summer—1,874; and Autumn—2,719 events, which translates to 9.7, 6.1, 4.3 and 5.8 $\times 10^{-5} \, \mathrm{s}^{-1}$, respectively.

**Table 1:** An overview of the the dwelling times and occurrence rates of MiMos during the four seasons

Season	Dwelling	Events	Occurrence
	Time (s)	#	Rate $\#/\text {s}$
Summer	$4.4 \times 10^7$	1874	$4.3 \times 10^{-5}$
Autumn	$4.7 \times 10^7$	2719	$5.8 \times 10^{-5}$
Winter	$6.3 \times 10^7$	6116	$9.7 \times 10^{-5}$
Spring	$4.9 \times 10^7$	2982	$6.1 \times 10^{-5}$

### Plasma-β

One of the drivers of the MiMo instability is the plasma-β, Eq. (1). Unfortunately, the FIPS data cannot be used to get an estimate of the thermal plasma pressure, due to the blocking of a significant part of the solar wind ions by the sun shield. Therefore, we use the data from the Tao solar wind propagation model (Tao et al. 2005) to get estimates of the solar wind plasma. For all four years discussed in this paper, we have taken the plasma density and temperature and the magnetic field and calculated the various pressures and plasma-β.

**Fig. 8:**
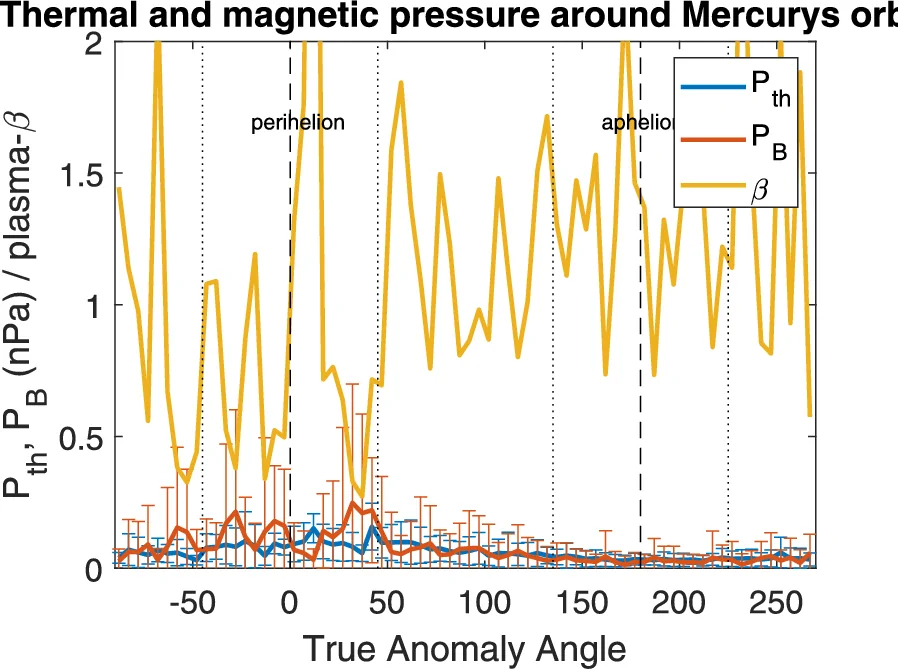
Thermal and magnetic pressure and the plasma-β around Mercury’s orbit, based on the Tao propagation model (Tao et al. 2005). The curves are the means of $5^{\circ }$ bins and the error bars are the standard deviations in the bins

The hourly plasma and magnetic field data from the Tao-model were smoothed with a sliding window with a size of 1 day. For each day the true anomaly angle of Mercury was determined (obtained from NAIF (The Navigation and Ancillary Information Facility, https://naif.jpl.nasa.gov/naif/.) The plasma and magnetic pressure and plasma-β were then binned in $5^{\circ }$ bins and the mean and standard deviation was determined, displayed in Fig. 8.

The obtained plasma-β of the solar wind at Mercury, based on the Tao-model, varies around a value of $\beta \approx 1$, with distinct β<1 conditions for $-75^{\circ } \le \mathrm{TAA} \le 50^{\circ }$ (around perihelion). This would point to ICWs dominating around perihelion and MiMos around aphelion. The variation in the plasma and magnetic pressure, described by the standard deviation within the angular bins (the error bars in Fig. 8) are much larger around perihelion (summer) than around aphelion (winter), and the average plasma-β is higher around aphelion than around perihelion. This difference can be explained by the more turbulent plasma and magnetic field closer to the Sun, and by the magnetic field changing less fast than the solar wind density radially away from the Sun.

Recently, Dewey et al. (2026) discussed the “seasonal effects” of the solar wind forcing at Mercury. Interestingly, they find (in their Fig. 2) that there is a difference in the location of maximum probability in maps of solar wind dynamic pressure ($P_\mathrm{dyn}$) versus Alfvén Mach number ($M_\mathrm{A}$) between perihelion (summer) and aphelion (winter). At aphelion there is a higher $M_\mathrm{A}$ and a lower $P_\mathrm{dyn}$.

Rewriting the plasma-β in terms of Mach number and dynamic pressure one finds:

6 $$\begin{aligned} \beta = \frac{2 n k T M_\mathrm{A}^2}{P_\mathrm{dyn}}. \end{aligned}$$

Using the values for highest probability from Dewey et al. (2026) and looking at the ratio $M_\mathrm{A}^2/P_\mathrm{dyn}$ and assuming that on average the solar wind density and temperature are relatively stable for perihelion and aphelion (see the slight variation in $P_\mathrm{th}$ in Fig. 8) we find with the aphelion centroid ($M_\mathrm{A} = 5.7, \, P_\mathrm{dyn} = 7.2$ nPa) and the perihelion centroid ($M_\mathrm{A} = 4.4, \, P_\mathrm{dyn} = 15.6$ nPa) that the ratio $M_\mathrm{A}^2/P_\mathrm{dyn}$ changes from 4.5 to 1.2 from aphelion to perihelion, which is a factor of 3.8. This agrees well with the arguments given above using the Tao-model.

## Regional differences

**Fig. 9:**
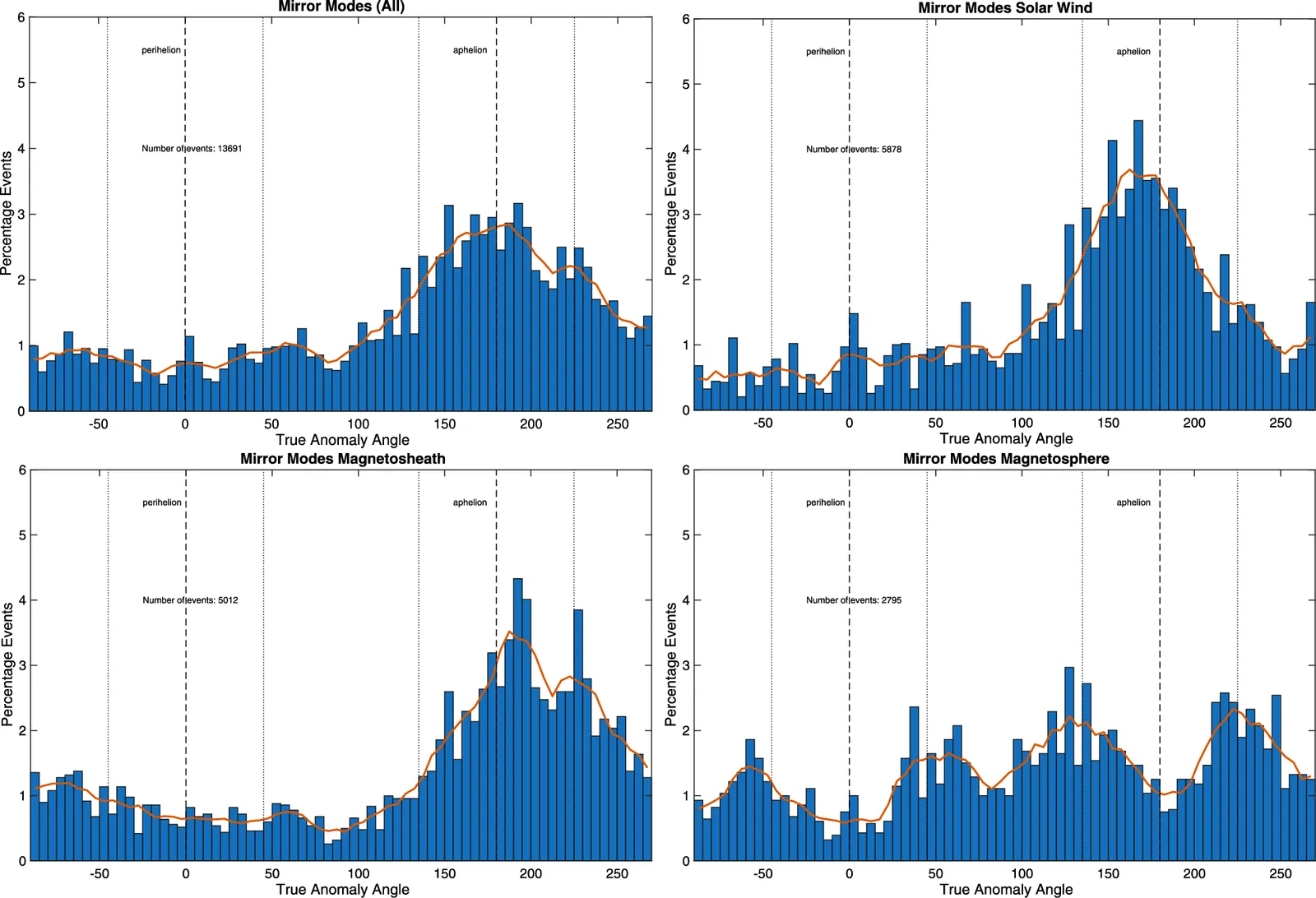
Histograms of the percentage of events per $5^{\circ }$ bins for MiMos (13,691 events). The red lines are sliding averages over 5 bins. Note that the number of events in three regions does not add up to the total of events as for 6 events no clear region could be assigned

It is clear from Fig. 6 that the events we have found are located in different regions around Mercury. They are present in either the solar wind, in the magnetosheath or in the magnetosphere. In order to determine in which region an event is located, we use the boundary crossings list by Philpott et al. (2020). In Fig. 9 we show the distribution of the MiMos as a function of true anomaly angle of Mercury, for all events and divided into the three regions. The distributions of the solar wind and magnetosheath MiMos are very similar. The MiMos in the magnetosphere, however, have a quite different distribution.

The similarities between the distributions in the solar wind and magnetosheath indicate that the conditions of the solar wind plasma being favourable for the generation of MiMos do not significantly change after crossing the Hermean bow shock. Also, MiMos, created in the solar wind, can be transported across the bow shock into the magnetosheath similar to MHs (Plaschke et al. 2018). Karlsson et al. (2022) showed Cluster observations of MHs crossing the Earth’s bow shock: first measured by Cluster spacecraft in the solar wind and later by Cluster spacecraft in the magnetosheath. Apparently, the MiMos will not cross Mercury’s magnetopause, considering the dip in the percentage of events around aphelion where the other two regions display a peak (Fig. 9).

Using the boundary crossings list by Philpott et al. (2020), we determine how much time MESSENGER dwelled in the three regions around Mercury: solar wind, magnetosheath and magnetosphere (as in Fig. 9). This will allow for the determination of the regional occurrence rate of the MiMos. We find the following values: overall $9.9 \times 10^{-5}$, solar wind $10.3 \times 10^{-5}$, magnetosheath $9.4 \times 10^{-5}$ and magnetosphere $0.8 \times 10^{-5}$ per second. This makes an interesting comparison with the occurrence rates for isolated MHs by Karlsson et al. (2021) mentioned above, the values are very close (solar wind $5 \times 10^{-5}$, magnetosheath $2 \times 10^{-4}$). This begs the question whether there is an overlap in the identification of MiMos and MHs.

An overview of all occurrence rates is given in Table 2.

**Table 2:** An overview of the occurrence rates of MiMos, MHs and ICWs in $\#/\text {s}$ and $\#/\mathrm{day}$

		All	Solar	Magneto-	Magneto	Venus	Mars
		regions	Wind	sheath	sphere	all regions	
MiMo	$\#/\text {s}$	$9.9 \times 10^{-5}$	$10.3 \times 10^{-5}$	$9.4 \times 10^{-5}$	$0.8 \times 10^{-5}$	$1.0 \times 10^{-3}$	$3.0 \times 10^{-3}$
	$\#/\mathrm{day}$	8.6	8.9	8.1	0.7	86	259
MH	$\#/\text {s}$	$2.6 \times 10^{-4}$	$5.0 \times 10^{-5}$	$2.0 \times 10^{-4}$	NA	–	$1.2^a \times 10^{-5}$
	$\#/\mathrm{day}$	22.5	4.3	17.3			10.4
ICW	$\#/\text {s}$	$6.0 \times 10^{-5}$	$5.4 \times 10^{-5}$	$8.0 \times 10^{-5}$	NA	$0.4/1.1^b \times 10^{-5}$	$3.5^c \times 10^{-5}$
	$\#/\mathrm{day}$	5.2	4.7	6.9		0.3/1.0	3.0

$^\text {a}$ Value from Madanian et al. (2020), MAVEN, June–September 2016.

$^\text {b}$ Values from Delva et al. (2015), Venus Express, 2 Venus years, lower value at solar minimum, higher value at solar maximum.

$^\text {c}$ Value from Wei et al. (2014), Mars Global Surveyor, obtained during the MGS AB1 phase

## Seasonal variations

It is clear from Fig. 7 that there are many more MiMos during winter time. In Table 1 it can be seen that the dwelling time in winter, i.e. around aphelion, is about 30% longer than for the other seasons, but the number of observed MiMo-intervals is more than double. However, we can also see that for Autumn and Spring there are dwelling gaps along the bow shock, resulting in gaps in the occurrence plots in the right column.

As discussed above, we assume that the MiMos are partly generated by pick-up ions from the Hermean exosphere. As these ions are picked-up by the IMF, they create a ring–beam distribution, which is unstable for the generation of MiMos and ion cyclotron waves (ICWs). Depending on the angle of the Parker spiral (Parker 1958), either the ring or the beam component of this inserted particle distribution will dominate. The more radial the field, the stronger the beam component, which will only excite the ion-beam–beam instability generating ICWs. When Mercury is around aphelion, the Parker spiral will have become slightly less radial, and thus more ring-component.

Another aspect of the increasing Parker spiral angle is that further from the Sun the region where there is a quasi-perpendicular bow shock increases, and thereby the possibility of energizing the ions perpendicular to the magnetic field as they cross the bow shock also increases. This can also add to the increase occurrence of MiMos at aphelion.

A third reason for the seasonal variation in the occurrence of MiMos can be found in the solar wind conditions along the orbit of Mercury. Fig. 8 shows that the variations in the magnetic and thermal pressures is much larger around perihelion (see the much larger error bars) than around aphelion. The “quieter” solar wind conditions around aphelion may be beneficial for the generation of MiMos.

## Mirror modes, magnetosonic waves or magnetic holes?

The magnetic-field-only method to identify MiMos naturally comes with limitations. There are two compressional waves, the slow and the fast magnetosonic waves, which could possibly be mis-interpreted as MiMos. One of the search parameters is the angle between the background magnetic field and the minimum variance direction, which is set to be $\phi _\mathrm{Bmv} > 70^{\circ }$ (see above). The minimum variance direction can also be seen as the propagation direction of the waves. The slow mode displays an anti-phase relationship between the magnetic field strength and the plasma density, just like the MiMo. However, the slow wave cannot propagate at large angles to the background magnetic field, and hence are not detected with this method. The fast waves have in-phase variations of the magnetic field strength and the plasma density, and can propagate in any direction. These waves can only be excluded using plasma data. Using MAVEN data, (Simon Wedlund et al. 2022, 2023) have discussed this detection ambiguity at Mars, pointing out that FM modes mostly occur in the close vicinity of the bow shock and immediately downstream of the magnetic pileup boundary, leaving MiMos to dominate in the magnetosheath. Consequently, as in those studies, we exclude those two specific regions from our dataset to ensure that the detected waves are most likely MiMos.

One topic that has not yet been discussed is mis-identification because of bow shock motion, like can be seen in Fig. 3. In order to estimate the number of possible mis-identifications we have used the boundary list by Philpott et al. (2020) and determined how many events were within a 2 min window from the bow shock, where the window size is based on the examples in Fig. 3. We find that out of the 13,691 events a total of 757 (or $\sim \, 5\%$) events could possibly be mis-identified.

As discussed above, MiMos and MHs are similar structures, characterized by a depression of the magnetic field strength and an increase in plasma pressure (dips), or, vice versa by an increase in magnetic field strength and a decrease in plasma pressure (peaks). Winterhalter et al. (1995) have shown, using Ulysses data, that solar wind MHs usually appear in MiMo-stable ambient plasma and posit that MHs may be the remnants of MiMos created upstream of the location of observation. MHs in the solar wind near Earth have been shown to be in pressure balance (Volwerk et al. 2021), however, as mentioned above, because of the instruments being behind the sun shield this cannot be checked for these structures at Mercury.

In order to check whether there can be confusion between the detection of MiMos and MHs in the MESSENGER data, we have analysed the magnetometer data in the same way as in Volwerk et al. (2020):

- The background magnetic field strength is determined by a sliding window average over 300 s: $B_{300}$;
- The data are smoothed by a sliding window average over 2 s: $B_{2}$;
- From the ratio time series $\Delta B/B_{300}$, lowest depressions are preselected that are separated by at least 300 s;
- The total magnetic field strength $B_{300} > 2$ nT;
- The depth of the structure $\Delta B/B_{300} = (B_{300} - B_{2}) / B_{300} > 0.5$.

**Fig. 10:**
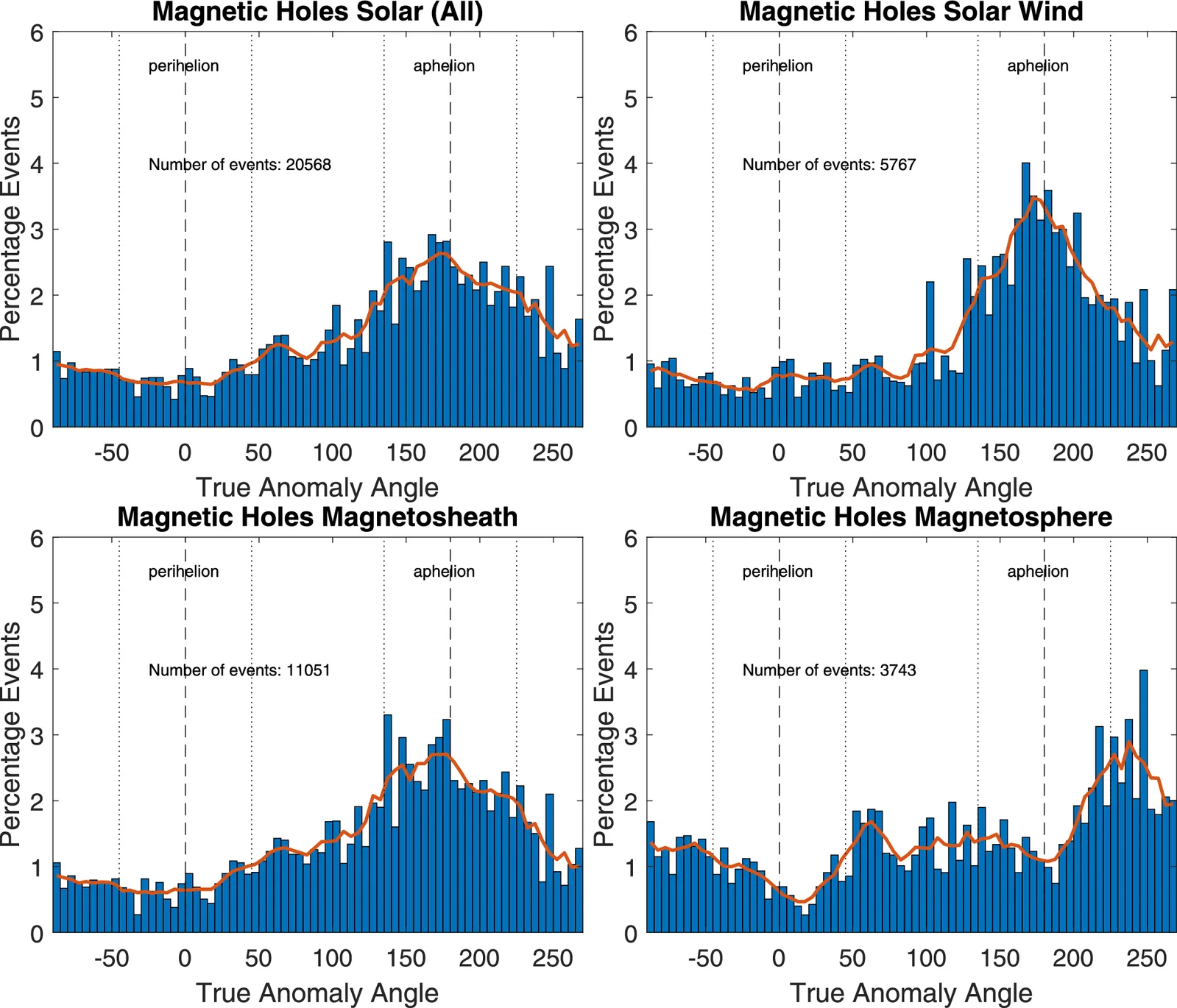
Histograms of the percentage of events per $5^{\circ }$ bins for linear MHs (20,568 events). The red lines are sliding averages over 5 bins

In order to comply with the results of Karlsson et al. (2021), we define the maximum magnetic field rotation over the MH as $\Delta \phi _\mathrm{max} = 25^{\circ }$ for the determination of so-called linear MHs. In total we find 20,568 MHs, which is approximately 50% more than the number of MiMos. Their distribution over the different regions and true anomaly angle is shown in the panels of Fig. 10. The occurrence rates of the linear MHs is: overall $2.1 \times 10^{-4}$, solar wind $9.1 \times 10^{-5}$, magnetosheath $6.4 \times 10^{-4}$ and magnetosphere $2.3 \times 10^{-4}$ per second. The rates are slightly higher than for the MiMos, but still on the same order of magnitude, and also slightly higher than the rates by Karlsson et al. (2021), which is consistent with the slightly different search criteria for MHs.

The panels in Fig. 10 show strong similarities with those in Fig. 9, which begs the question about the identification of either structure. Therefore, we take the time intervals of the MiMos and compare them with those of the MHs. The length of the MiMo intervals goes up to 250 s (Fig. 2). We search for MHs which have their central time (minimal *B*) within a window around the MiMo intervals. This results in an overlap of 4299 MiMos and MH, which means $\sim \, 40\%$ of the MiMos might be mis-identified.

**Fig. 11:**
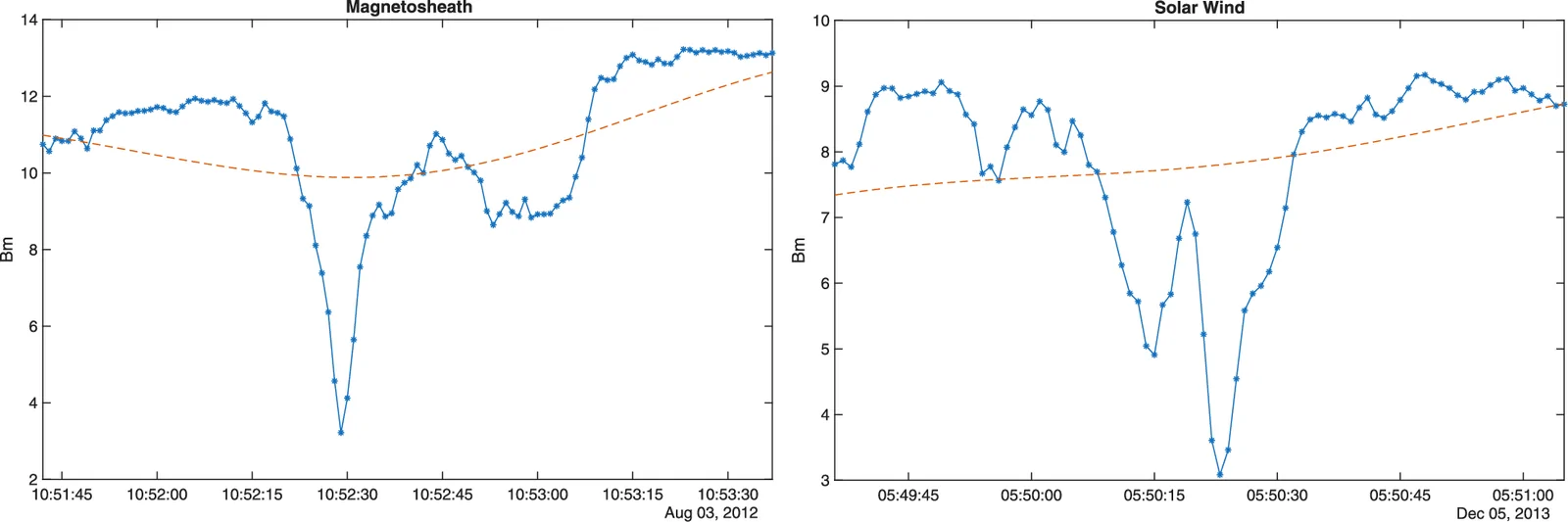
Two examples of doubly identified structures. These two are both found through the mirror-mode and the magnetic hole search algorithms

There is a slight difference between the three regions: solar wind:magnetosheath:magnetosphere = 1,884:1,402:1,013 = 32%:36%:44%. Karlsson et al. (2021) also investigated MHs around Mercury using the MESSENGER data. In their analysis it is also clear that the search algorithms have difficulty distinguishing between MHs and MiMos (see, e.g., their Figs. 2 and 3). Two examples of doubly identified structures are shown in Fig. 11.

## Comparison with solar wind ion cyclotron waves

**Fig. 12:**
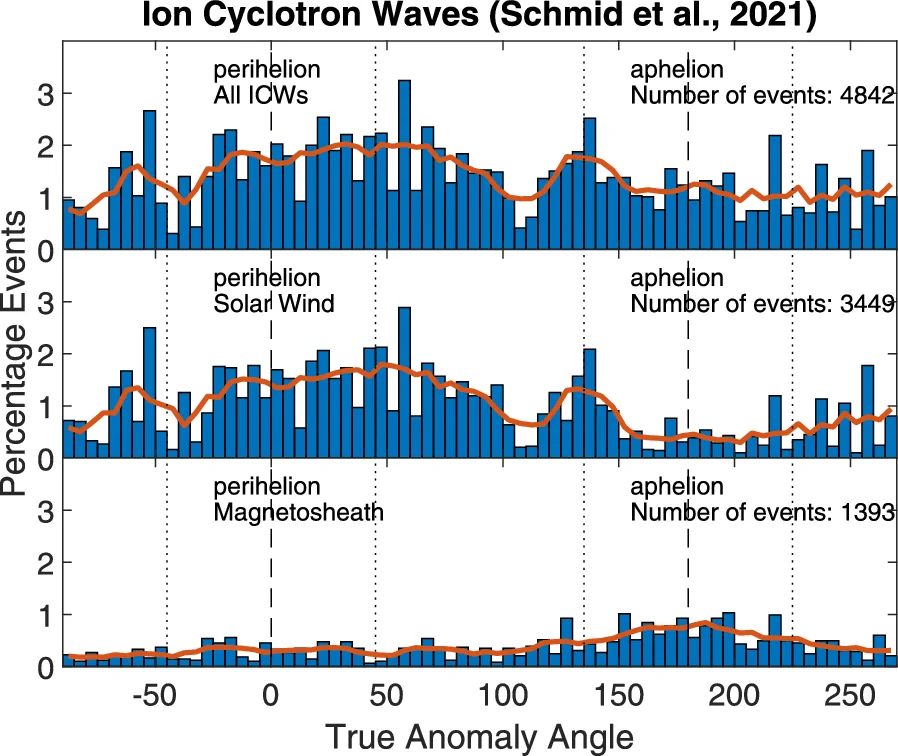
Histograms of the percentage of events per $5^{\circ }$ bins for the ICWs detected by Schmid et al. (2021) for ${\textbf{H}^{+}}$. From top to bottom are shown: all ICWs, solar wind ICWs (normalized by total IWCs) and magnetosheath ICWs (normalized by total IWCs). The red lines are sliding averages over 5 bins

We compare this seasonal effect on the MiMos with that on the Ion Cyclotron Waves (ICWs) detected by the MESSENGER magnetometer (Schmid et al. 2021, 2022) for the years 2012 through 2015 in the solar wind ($\sim \, 73\%$) and the magnetosheath ($\sim \, 27\%$). Histograms of the percentage of events as a function of the true anomaly angle of Mercury are shown in Fig. 12. The top panel shows the distribution of all ICWs as a function of the true anomaly angle, which looks more constant in comparison with the distributions of the MiMos and MHs. Splitting up between solar wind and magnetosheath shows an asymmetry. In the solar wind there is a decrease around aphelion, whereas in the magnetosheath there is a slight increase around aphelion. The occurrence rates for ICWs are: overall $5.0 \times 10^{-5}$, solar wind $5.4 \times 10^{-5}$ and magnetosheath $8.0 \times 10^{-5}$ per second.

## Discussion and conclusions

We have studied the MESSENGER magnetometer data for the years 2011 through 2015 (corresponding to 16.5 Hermean years) to find evidence, or lack thereof, for the presence of mirror modes. The detection method used magnetic field data only, because of the poor time resolution of the FIPS plasma instrument onboard MESSENGER and the fact that part of the particle distribution is blocked from the sensor by the heat shield. However, the FIPS data could be used to place the detected events in context.

In total 13,691 intervals of 20 s or longer were found, both in the solar wind and in Mercury’s magnetosheath and magnetosphere. Because of the average Parker-spiral angle $\phi _\mathrm{Pa} \approx 25^{\circ }$ (Chang et al. 2019) much of the Hermean bow shock will be quasi-parallel, i.e. where the magnetic field and bow shock normal are at a small angle with respect to one another. MiMos are usually expected to appear behind a quasi-perpendicular bow shock, where the temperature anisotropy can be created by enhanced gyratory motion of the ions caused by (multiple) reflection(s) at the bow shock before entering the magnetosheath shock drift acceleration, see, e.g., (Kirk et al. 1994, chapter 2.2) as well as energization through the compression of the magnetic field combined with the conservation of the first magnetic moment. The MiMos in the Hermean magnetosheath, however, are present both behind the quasi-perpendicular and quasi-parallel bow shock. This means that the MiMos are less created by energization of the ions when they cross the bow shock, but more likely the result of ion pick-up in the extended Hermean exosphere. This process also takes place near Mars (Simon Wedlund et al. 2025).

The spatial distribution of MiMos in Mercury’s environment is similar to the distributions around Venus (Volwerk et al. 2023) and Mars (Simon Wedlund et al. 2023), but their occurrence rate is almost 2 orders of magnitude less as compared with Venus and Mars.

There is a strong variation of the occurrence rate of MiMos with the seasons of Mercury. In the winter season, around aphelion, there are many more MiMo detections than in summer, around perihelion. A histogram showing the percentage of events as a function of Mercury’s true anomaly angle clearly shows a preference for MiMos around aphelion. Similarly, the distribution of MHs also shows a peak near aphelion. A histogram of the ICWs measured around Mercury (Schmid et al. 2021) shows that these waves are less sensitive to Mercury’s true anomaly angle, but have a maximum around perihelion in the solar wind, however they show a slight maximum around aphelion within the magnetosheath.

Using the Tao solar wind propagation model (Tao et al. 2005), we show that the variations in the magnetic field strength and the plasma density are much smaller at aphelion than at perihelion. It should be noted that the solar wind plasma-$\beta \lesssim 1$ around Mercury’s perihelion, usually unfavourable for MiMo generation (see also Fig. 1), whereas $\beta \ge 1$ around Mercury’s aphelion, more favourable for MiMo generation.

When ion pick-up is the main source for the generation of ICWs and MiMos see e.g., (Simon Wedlund et al. 2022), creating a ring–beam distribution into the solar wind, the asymmetry along Mercury’s orbit may suggest that MiMo generation is favoured in more stable magnetic field conditions, whereas ICWs benefit from larger B-field variations in order to resonate with the background wave spectrum.

We conclude that:

- Mirror modes do exist near and inside of Mercury’s magnetosheath;
- Mirror modes are most prone to be generated around Hermean aphelion;
- Mirror modes and magnetic holes have similar dependence on true anomaly angle;
- Pick-up ion cyclotron waves have a lesser dependence on true anomaly angle.

With the upcoming two-spacecraft mission BepiColombo (Anselmi and Scoon 2001; Benkhoff et al. 2010; Benkoff et al. 2021) a better understanding of the occurrence of MiMos, MHs and ICWs will be obtained, especially during period when the Mercury Planetary Orbiter (MPO) is measuring the planetary environment and the Mercury Magnetospheric Orbiter (*Mio*) makes in situ measurements of the solar wind conditions.

## Data Availability

All MESSENGER data are available from NASA’s Planetary Data System (PDS), https://pds.nasa.gov/. The Tao solar wind propagation model can be accessed through AMDA, https://amda.irap.omp.eu/.

## Footnotes

1 Brazil plot: a plot of the plasma temperature anisotropy $T_{\perp }/T_{\Vert }$ versus the plasma-β of a set of magnetoplasma data, usually with the marginal stability curves of the ion cyclotron, mirror mode and (oblique) firehose instabilities drawn in. The distribution of the data points usually spreads out in the shape of the country Brazil, hence the name of the plot.

2 TAA: true anomaly angle, the angular position of Mercury in its orbit around the Sun, measured from the planet’s perihelion.

3 MSO: Mercury Solar Orbital coordinate system, the *X*-axis points to the Sun, the *Y*-axis points anti-parallel to Mercury’s orbital velocity and the *Z*-axis completes the right-hand coordinate system.

4 $1 R_\mathrm{M}$ = 1 Mercury radius = 2439.7 km
